# Can ethical artificial intelligence aid infant and young child feeding support during conflict? A review of the experience and lessons learned from FHI 360′s in Ukraine

**DOI:** 10.3389/fnut.2026.1745609

**Published:** 2026-03-31

**Authors:** Alessandro Iellamo, Olena Rozhenko, Yuliia Strelchenko-Yankovska, Nataliia Kalashnyk, Inna Burta, Celestine Ekwuluo, Yelyzaveta Hryshyna, Oleksandra Firsova, Vitalii Sheremet

**Affiliations:** 1 FHI 360, Crisis Response and Resilience, Washington, DC, United States; 2 FHI 360 Nutrition, Crisis Response and Resilience Department, Dnipro, Ukraine

**Keywords:** artificial intelligence for health and nutrition, breastfeeding counseling, breastfeeding in emergencies, breastfeeding support, complementary feeding in emergencies, conflict-affected health and nutrition services, digital humanitarian assistance, infant and young child feeding in emergencies

## Abstract

**Background:**

The war in Ukraine has disrupted access to maternal and infant nutrition services, intensified commercial milk formula marketing risks, and increased demand for accessible, evidence-based breastfeeding support. To respond, FHI 360 and UNICEF Ukraine deployed *Harmony of Parenthood*, a closed-domain, ethically governed Artificial Intelligence (AI)-enabled breastfeeding and Infant and Young Child Feeding in Emergencies (IYCF-E) support system, integrated with facility-based counseling and a national mentorship programme for lactation counselors.

**Objectives:**

The aim of this study was to evaluate the feasibility, safety, acceptability, and operational contribution of an AI-enabled hybrid counseling model to continuity and quality of Infant and Young Child Feeding in Emergencies services during protracted conflict, including its integration with facility-based counseling and mentorship systems and its potential influence on caregiver feeding practices and counseling quality.

**Methods:**

A convergent mixed-methods approach triangulated: (1) chatbot analytics (user characteristics, interaction volume, response quality); (2) facility counseling data from four conflict-affected oblasts; (3) structured competency assessments following >600 mentorship visits; and (4) qualitative feedback from caregivers and health workers. Governance, safety, and Code-compliance safeguards were assessed against the World Health Organization (WHO) AI ethics guidance, the Operational Guidance on Infant and Young Child Feeding in Emergencies (OG-IFE), and Ukraine's breastfeeding policies.

**Results:**

Between November 2024 and October 2025, 2,066 caregivers generated more than 38,000 chatbot interactions. Ninety-eight percent of answers met accuracy and safety criteria; one hallucination event was detected and corrected through real-time review. Caregivers used the system predominantly during periods of insecurity or when services were inaccessible, valuing its 24-h availability, emotional reassurance, and escalation to human counselors. In parallel, facility data showed improved quality of counseling, strengthened referral pathways, and increases in early initiation and exclusive breastfeeding among women receiving repeated support. Mentorship visits demonstrated competency gains among lactation counselors, enhanced adherence to the Code, and more consistent use of MoH-aligned counseling tools.

**Conclusion:**

An AI-enabled, human-supervised hybrid model is feasible, acceptable, and safe for sustaining breastfeeding and IYCF-E support during active conflict. When anchored in authoritative guidance and embedded within national systems, AI tools can complement skilled counselors, strengthen continuity of care, and uphold Code-compliant, evidence-based support for mothers and infants. To our knowledge, this is the first documented evaluation of an AI-enabled IYCF-E intervention implemented during an active conflict.

## 1 Introduction

Optimal breastfeeding remains one of the most effective interventions for improving child survival, maternal health, and long-term human capital. Near-universal breastfeeding could prevent an estimated 823,000 child deaths and 20,000 maternal deaths annually through reductions in infectious morbidity and maternal cancers (1–3). Breast milk provides complete nutrition and immunological protection against diarrhea, pneumonia, neonatal sepsis, and other leading causes of infant morbidity and mortality (1, 3). The *Lancet* Breastfeeding Series further highlights cognitive, metabolic, and developmental benefits that extend into adulthood (1, 3, 4). Economically, premature cessation of breastfeeding is associated with global losses of approximately US$302 billion annually, reinforcing breastfeeding as a critical public health and economic priority (5).

In emergencies, breastfeeding becomes even more vital. Disruptions to health systems, water and sanitation, household food security, and psychosocial support substantially increase infant vulnerability. Evidence from diverse crises consistently shows that non-breastfed infants have a six- to twenty-five-fold increased risk of mortality due to diarrhoeal disease and respiratory infections (6). Uncontrolled distribution of commercial milk formula (CMF) during emergencies, documented, for example, after the 2006 Yogyakarta earthquake, has been linked to significant rises in infant diarrhea and malnutrition (7). These risks underscore the relevance of global normative guidance, including the International Code of Marketing of Breast-milk Substitutes (8) and the Operational Guidance on Infant and Young Child Feeding in Emergencies (OG-IFE) (9), which outline essential standards for breastfeeding protection, skilled support, Code-compliant communication, and coordinated multisectoral action.

Despite more than two decades of consensus, major implementation gaps persist. Analyses of emergency responses highlight insufficient preparedness, inadequate counseling capacity, limited coordination, and inconsistent adherence to the Code and OG-IFE across crisis-affected countries (10–15). Exclusive breastfeeding (EBF) remains far below global targets, with only 48% of infants under 6 months exclusively breastfed compared to the 2030 target of 70% (16, 17). These deficits leave caregivers exposed to misinformation, conflicting advice, stress-related breastfeeding concerns, and exploitative marketing practices that undermine optimal feeding.

Ukraine represents a critical context where these systemic vulnerabilities converge. Even prior to the full-scale escalation of war in 2022, exclusive breastfeeding rates were suboptimal despite extensive Baby-Friendly Hospital Initiative (BFHI) certification (18). The conflict has caused widespread damage to health infrastructure, displacement of health workers, fragmentation of referral pathways, and reductions in routine maternal and newborn care (19, 20). FHI 360′s qualitative assessment conducted in 2022 documented that psychological stress, insecurity, displacement, and misinformation profoundly disrupted breastfeeding intentions and practices, with many women reporting pressure from health workers or volunteers to introduce CMF and receiving unsolicited CMF donations practices that contravene both the Code and OG-IFE (21). These findings highlighted substantial gaps in skilled counseling, continuity of care, facility-level capacity, and regulatory oversight, underscoring the need for innovative, resilient approaches to infant and young child feeding during protracted conflict.

Digital technologies offer important opportunities to support continuity of care, expand access to skilled counseling, and strengthen health system resilience during emergencies, particularly for maternal, newborn, and child health services in fragile and conflict-affected settings. Global guidance from the World Health Organization and UNICEF highlights the critical role of digital health interventions in improving access, quality, and continuity of care where conventional service delivery is disrupted (22, 23). Concurrently, digital health and artificial intelligence (AI) offer emerging opportunities to strengthen access to timely, evidence-based counseling for caregivers in crisis settings. Systematic reviews show that digital and AI-enabled health tools can improve maternal knowledge, service utilization, adherence to recommended practices, and continuity of care across diverse contexts (24–27). Despite this potential, the authors did not find any published studies that have documented the use of AI-enabled or chatbot-based interventions specifically targeting infant and young child feeding in humanitarian settings, representing a significant evidence gap at the intersection of AI, digital health, and emergency nutrition support. Existing chatbots used by humanitarian agencies largely focus on general information provision, mental health support, or administrative assistance rather than structured, Code-compliant, OG-IFE-aligned breastfeeding counseling (28–30).

In response to these needs, FHI 360 launched Harmony of Parenthood in 2024, an AI-enabled, human-supervised counseling chatbot providing 24/7 support on breastfeeding, complementary feeding, maternal well-being, and psychosocial concerns to caregivers across conflict-affected oblasts of Ukraine. Built on a controlled knowledge-base derived from WHO, UNICEF, IFE Core Group, and Ministry of Health guidance, the system integrates automated responses with direct referrals to trained lactation counselors, IYCF focal persons, and psychologists for complex queries. All content was technically validated and adapted for the Ukrainian context, aligned with FHI 360′s AI Artificial Intelligence Use and Governance policy (31) and WHO guidance on ethical AI in health (32). The chatbot functions as part of a broader IYCF-E programme, including facility-based IYCF-E training, structured mentorship and supervision, peer-support groups, and strengthened referral pathways, implemented by FHI 360 with UNICEF support.

This hybrid digital–human model operationalizes core OG-IFE recommendations by:

1. providing accessible, accurate, Code-compliant breastfeeding information;
2. enabling continuous, on-demand support when in-person services are disrupted;
3. integrating psychosocial support within IYCF counseling;
4. linking digital platforms to trained human counselors and health facilities; and
5. strengthening referral systems and continuity of care.

This study presents the first mixed-methods evaluation of an AI-enabled IYCF-E intervention implemented during an active armed conflict. This study therefore, aimed to evaluate the feasibility, safety, acceptability, and operational contribution of the AI-enabled hybrid counseling model within the broader IYCF-E programme during active conflict.

Given these gaps and the urgent need for continuity of skilled breastfeeding support during the war, we adopted a mixed-methods framework to assess the feasibility, safety, and contribution of the AI-enabled hybrid model within real-world health system constraints.

## 2 Materials and methods

### 2.1 Study design and period

This study used a descriptive mixed-methods design to assess the feasibility, performance, accuracy, and acceptability of Harmony of Parenthood, an artificial intelligence (AI) enabled counseling chatbot integrated within FHI 360′s Infant and Young Child Feeding in Emergencies (IYCF-E) programme in conflict-affected oblasts of Ukraine. Mixed methods were necessary to triangulate user analytics, counseling quality, and human perspectives in a rapidly evolving crisis setting the evaluation covered the period from November 2024 to October 2025, corresponding to the third year of the full-scale war.

Quantitative analyses captured user reach, engagement patterns, thematic query distribution, responsiveness, and referral flows. Qualitative data provided insight into user experience, trust, and perceived value. Facility-based counseling and mentorship data contributed to triangulation across digital and in-person support systems.

The evaluation was coordinated by FHI 360′s Crisis Response and Resilience Department and aligned with the World Health Organization (WHO) guidance on Ethics and Governance of Artificial Intelligence for Health (32).

### 2.2 Study setting and context

The intervention was implemented in Dnipro, Mykolaiv, Odesa, and Zaporizhzhia. These oblasts experienced repeated attacks, population displacement, and disruptions to maternal and newborn health services. Many primary health facilities temporarily closed or reduced service provision due to insecurity, staffing shortages, or infrastructure damage, limiting access to skilled lactation support and psychosocial care.

Harmony of Parenthood was designed as a 24-h Ukrainian-language digital counseling platform operating alongside a broader IYCF-E programme that included facility-based training, structured mentorship and supervision, digital mother-to-mother support groups, Code-compliant information, and referral mechanisms. This integrated approach operationalised OG-IFE recommendations including safe communication channels, skilled counseling, cross-sector coordination, and psychosocial support.

### 2.3 Chatbot architecture and workflow

The system architecture consisted of Telegram, SendPulse, OpenAI, and Canva.

Telegram functioned as the encrypted user interface for structured and free-text queries. Users joined the chatbot (@HarmonyParentBot) via QR codes, links, or keyword search. Users accessed the chatbot through multiple entry points, including QR codes displayed in participating health facilities, targeted social media promotion, and direct referral by trained lactation counselors during antenatal, postnatal, and infant feeding consultations. Once enrolled, users could interact with the chatbot via structured menu navigation or free-text queries at any time. Examples of user prompts included questions such as: “My baby is not latching properly, what should I do?”, “How can I increase my milk supply?”, and “When should I start complementary feeding?” The chatbot provided structured, evidence-based responses aligned with WHO and national infant feeding guidance, including practical recommendations and referral to trained lactation counselors when additional support was needed.

The system automatically recorded anonymised interaction metadata, including timestamps, session duration, query type, navigation pathway, automated referral triggers, and chatbot response outputs. These interaction logs enabled analysis of user engagement patterns, thematic distribution of caregiver concerns, system responsiveness, retention, and referral linkages between digital interactions and facility-based counseling services. In addition, response accuracy and safety were continuously monitored through structured review of chatbot outputs against the validated knowledge base, allowing identification and correction of any inaccuracies or hallucinations.

SendPulse served as the orchestration layer, managing conversation trees, tone-of-voice parameters, automated flows, and fallback messages. It also enforced content restrictions prohibiting medical prescriptions, diagnostic guidance, or any reference to commercial brand names, in accordance with the International Code of Marketing of Breast-milk Substitutes (8).

OpenAI generated real-time responses through secure API keys. The chatbot relied on a controlled knowledge base containing validated entries extracted from WHO, UNICEF, the IFE Core Group, and Ministry of Health of Ukraine guidance. Structured prompts constrained the AI to infant feeding, maternal nutrition, pregnancy, early childhood care, and psychosocial topics. All originally English-language materials were translated and culturally adapted for the Ukrainian context and validated by FHI 360′s Crisis Response and Resilience Nutrition Team.

Canva was used to create static and animated educational visuals embedded within conversation flows and shared across digital outreach channels.

Together, these components formed a hybrid digital–human architecture designed for accuracy, safety, scalability, and continuous oversight.

### 2.4 AI configuration, accuracy assurance, and hallucination management

The chatbot operated on a closed-domain knowledge base to avoid unverified information. Structured prompts defined the boundaries of all outputs. Weekly log reviews by technical specialists identified inconsistencies, prompted refinement of instructions, and informed updates to the knowledge base.

Hallucinations were defined as incorrect or fabricated responses generated by the AI that are not supported by authoritative sources (33). These were mitigated through fallback responses instructing the chatbot to acknowledge when information was unavailable rather than speculate. Only one hallucination occurred during the evaluation period and was corrected within 24 h.

The oversight process followed WHO's guidance on the ethical governance of AI in health (32).

### 2.5 Participants and inclusion criteria

Digital participants included all users who interacted with the chatbot between November 2024 and October 2025. The chatbot was used by pregnant women and caregivers of infants and young children across conflict-affected oblasts. Demographic information such as age group, caregiver type, and oblast was optional and fully anonymised. No personally identifiable information or geolocation data were collected.

Access to the chatbot required a smartphone with internet connectivity and the Telegram messaging application. Telegram is widely used across Ukraine and remained accessible in most conflict-affected oblasts throughout the study period. Users could access the chatbot via QR codes displayed in participating health facilities, direct referral by trained lactation counselors, or social media outreach. Despite extensive infrastructure disruption caused by the war, national telecommunications monitoring and international data indicate that internet penetration in Ukraine exceeds 75%, and mobile cellular subscriptions exceed 130 per 100 inhabitants, reflecting widespread smartphone ownership and mobile connectivity even in conflict-affected regions (34, 35). Telecommunications providers prioritized restoration of mobile networks, allowing caregivers to maintain access to digital communication tools and health information platforms. These conditions enabled eligible caregivers to access and interact with the chatbot, ensuring feasibility of participation despite insecurity, displacement, and disruption of facility-based services.

Facility-level participants included 100 and 45 trained IYCF focal persons and mentors who completed counseling and mentorship forms through KoboToolbox. Verbal consent was obtained prior to data collection.

### 2.6 Data sources and collection procedures

Three data streams were used for analysis.

Digital analytics from Telegram and SendPulse included the number of unique users, incoming and outgoing messages, total interactions, response ratios, bounce rates, retention rates, average questions per session, topic categories, and automated referral flags. User feedback was collected using optional post-interaction surveys that assessed satisfaction, usefulness, clarity, and likelihood of reuse, along with open-ended comments. Facility-based counseling and mentorship data were collected with two KoboToolbox instruments. The Mentorship and Coaching Checklist recorded performance across 44 indicators including counseling accuracy, empathy, communication, use of behavioral-change materials, and integration of psychosocial messages. The Counseling Session Form documented presenting concerns, infant-feeding practices, agreed counseling solutions, and medical or psychosocial referrals. All tools were pretested to ensure clarity and logical consistency. Facility identifiers enabled mapping of chatbot referrals to follow-up counseling.

### 2.7 Outcome measures

The evaluation examined predefined outcomes reflecting the structure of the hybrid digital and facility-based intervention.

User volume and engagement included total unique users, number of interactions, user-initiated messages, repeat sessions, distribution by age and oblast, and the relative use of structured menus vs. free-text queries.

Retention, bounce, and responsiveness metrics described platform performance. Retention measured return users. Bounce rate represented the proportion of users who opened the chatbot but did not perform any action. Responsiveness was calculated as the ratio of chatbot replies to user messages.

Counseling quality indicators came from mentorship visits and included adherence to IYCF-E standards, completeness of feeding assessments, provision of practical breastfeeding support, use of communication and IEC materials, and integration of mental-health considerations into counseling sessions.

Exclusive breastfeeding was measured for mothers who received two or more counseling sessions. Feeding practices at initial and follow-up encounters were compared to assess changes in exclusive breastfeeding, mixed feeding, and formula feeding.

Mental health and psychosocial support referrals were documented through both chatbot referral prompts and facility-based records and included referrals for psychological support, pediatric care, obstetric and gynecological services, and rehabilitation.

Mentorship competency scores recorded progressive improvements across the 44 indicators assessed at each mentorship visit. Competencies included analysis of feeding difficulties, empathetic communication, practical breastfeeding support, use of behavior change materials, and integration of psychosocial support into routine counseling.

### 2.8 Data analysis

Quantitative data from SendPulse and KoboToolbox were analyzed using Microsoft Excel 365 for cleaning and indicator generation. Pivot tables were used for cross-tabulation by oblast and reporting period. Additional analyses including frequency distributions and comparisons of feeding practices at baseline and follow-up were conducted using SPSS Version 11. Qualitative data from user comments and counselor notes were analyzed using reflexive thematic analysis following the approach described by Braun and Clarke (36, 37). Codes were developed inductively and refined through iterative team review.

A convergent mixed-methods approach was used following the methodology described by Creswell and Plano Clark (38), enabling the integration of quantitative and qualitative findings across digital and facility-based data streams.

To strengthen analytical rigor, a convergent mixed-methods triangulation approach was applied, integrating chatbot analytics, facility-based counseling data, structured mentorship competency assessments, and qualitative user feedback. This approach enabled comprehensive evaluation of system performance, user engagement, counseling quality, and the operational contribution of the hybrid digital–human model to sustaining Infant and Young Child Feeding in Emergencies (IYCF-E) services during conflict.

### 2.9 Ethical considerations

All procedures adhered to FHI 360′s Global Data Privacy and Protection policy, and WHO guidance on AI ethics (32, 39). Only anonymized, aggregated data were used in the analysis. No personally identifiable information was collected.

The activity was reviewed by FHI 360′s Office of International Research Ethics and designated as non-human-subjects operational research. Verbal consent was obtained prior to facility-based data collection.

### 2.10 Data security and quality assurance

All data transfers between Telegram, SendPulse, OpenAI, and KoboToolbox were encrypted using HTTPS and TLS protocols. Access controls relied on multi-factor authentication and role-based permissions.

Quality assurance measures included automated skip patterns and logic checks within KoboToolbox, weekly review of submissions by monitoring and evaluation teams, monthly reconciliation between chatbot referrals and facility-based follow-up, and continuous dashboard monitoring by programme teams. All procedures complied with national and international data protection regulations including the General Data Protection Regulation.

## 3 Results

### 3.1 User demographics

Between November 2024 and October 2025, the *Harmony of Parenthood* chatbot reached 2,066 unique users, most of whom were women of reproductive age. Age distribution was < 18 years (1 %), 18–29 years (57 %), 30–44 years (37 %), and ≥ 45 years (5 %). This pattern aligned closely with the target population, pregnant and lactating women and caregivers of infants and young children.

A smaller proportion of older users, probably grandmothers or extended family members, demonstrated the platform's ability to influence household-level feeding decisions. Younger users frequently mentioned the anonymity and accessibility of Telegram, particularly during periods of curfew or displacement when travel to facilities was restricted.

Geographically, users were located across all oblasts, with highest concentrations in Odesa, Mykolaiv, Dnipro and Zaporizha mirroring both population density and the presence of **FHI 360-supported health facilities** with trained lactation counselors. These results confirm effective targeting through Telegram communities, social-media promotion, and counselor referrals.

The study population comprised all individuals who interacted with the chatbot between November 2024 and October 2025 and therefore represents a complete census of chatbot users during the evaluation period rather than a sampled subset. Eligibility was defined by voluntary interaction with the chatbot and did not require enrolment through health facilities or programme registration. As such, the denominator reflects all users who accessed the platform, rather than the total population of pregnant women and caregivers residing in the intervention areas. Participation depended on caregiver awareness of the chatbot, access to a smartphone and internet connectivity, and willingness to engage with digital support. Consequently, chatbot users may not be fully representative of all caregivers in conflict-affected oblasts, particularly those with limited digital access, lower digital literacy, or reduced exposure to programme outreach. These considerations should be taken into account when interpreting user characteristics and engagement patterns, and the findings should be understood as representative of the population of caregivers who accessed the digital intervention rather than the entire underlying population of eligible caregivers.

### 3.2 Platform performance indicators

Platform analytics from November 2024–October 2025 confirm high engagement and technical reliability (Table 1).

**Table 1:** *Harmony of parenthood* performance indicators.

Indicator	Value	Definition/Meaning
Total number of unique users	2,066	Users who have subscribed to the chatbot.
Total incoming messages	17,496	Users initiated interactions.
Response volume	20,842	Total chatbot answers provided.
Number of interactions	38,338	Total chatbot—user conversational events.
Bounce rate (%)	32 %	Bounce Rate (%)—the percentage of sessions where users opened the chatbot but did not take any action (did not click a button, open a topic, or reach advice). < 20%: Excellent—most users interact actively. 20%−40%: Good—engagement is stable, some users drop off. 40%−60%: Moderate—needs improvement in user flow. >60%: High bounce—many users leave without interaction.
Retention rate (%)	104%	Retention Rate (%)—the percentage of users who returned to the chatbot compared to the previous period. >100%: Growth—new users joined in addition to returning ones. 60%−100%: Good—most users came back. 30%−60%: Moderate—partial user return. < 30%: Low—few users returned.
Avg. questions per session	5	This indicator shows the average number of user questions per session. On average, users ask 2–3 questions per chat, often on different topics. The value reflects all messages per session, not only unique or unanswered ones. 1–2 Very effective—users find answers quickly. 3–5 Optimal—clear responses and good engagement. 6–8 Users may struggle; simplify structure or menu. >8 Too many steps—bot may confuse users.
Response rate	1.17	**Good**: Ratio ≥ 1.0 (i.e. bot replies to all user messages, and sometimes sends an additional follow-up) **Acceptable**: Ratio 0.8 to < 1.0 (bot replies to most messages, but some go unanswered) **Needs improvement**: Ratio < 0.8 (many incoming user messages get no bot reply)
User satisfaction (*n* = 266)	93% satisfied; 97% useful; 99% clear; 93% intend to reuse	Acceptability and usability metrics.

### 3.3 Reach and retention

During the 11-month period, the chatbot recorded nearly 40,000 interactions. Interactions rose from 9,362 in Q1 to 10,631 in Q4, while retention improved from 0% to 170%. The 32% bounce rat**e** shows that the majority of every initiated chat resulted in a complete exchange. These trends reflect expanding awareness and trust, reinforced by counselor referrals and supported digital outreach.

### 3.4 System responsiveness and reliability

The response rate is above 1 (1.17), meaning that after several optimizations and prompts and scenario logic the chatbot always provides more than one message/information based on the question received. No major outages occurred; one minor bug was fixed within 24 h. Continuous monitoring and human oversight maintained seamless operation.

### 3.5 User satisfaction and experience

In October 2025, among 266 respondents to user-experience surveys, 93% rated the chatbot satisfactory or very satisfactory, 97% found information useful, 99% found responses clear, and 93% said they would reuse it.

“I used the chatbot, I figured out the functionality quickly, just entered a keyword. For simple cases, especially if it's the middle of the night and no one else can advise, it's a very useful bot.” (Mother, Odesa Oblast)

“Thank you for developing the Harmony of Parenthood chatbot.” (Mother, Mykolaiv Oblast)

“Very cool functionality, a lot of prepared information—you can read or watch a video. Everything is clear. If there's no time to search, you can just write your question and get an answer quickly. Most importantly, you can sign up for a consultation and get free qualified advice. I'm glad that in Ukraine they care about nursing mothers.” (Mother, Dnipro Oblast)

“Yesterday I needed to find information on why babies often ask for breasts in the first months… it was easy and convenient to find, and the answer came immediately. With a small child, this is the salvation of motherhood! Thank you for supporting moms from all sides.” (Mother, Kharkiv Oblast)

These reflections show that users perceived the chatbot as empathetic, trustworthy, and lifesaving, bridging emotional reassurance with access to professional guidance.

### 3.6 Query distribution and thematic trends

Frequent topics included breastmilk-supply concerns (40%), pregnancy diet (39%), anxiety and sleep (38%), emotional regulation (36%), caffeine use during breastfeeding (35%), and how to manage a crying baby (33%).

### 3.7 Integration within FHI 360-supported health facilities

The chatbot was embedded within FHI 360-supported oblast health facilities (123), implemented with technical and financial support from UNICEF Ukraine. It complemented face-to-face counseling in Odesa, Mykolaiv, Dnipro, and Kharkiv. Complex queries were triaged to trained lactation counselors for follow-up via Telegram or in person.

The intervention was closely coordinated with regional and oblast health offices. Periodic analytic dashboards informed adaptive messaging and training.

### 3.8 In person field-based IYCF training, mentorship and counseling

#### 3.8.1 Scale and scope

The FHI 360 model, supported by UNICEF, introduced a structured capacity-strengthening approach to improve IYCF-E practices and services. The approach combined comprehensive technical training with facility-based mentorship and ongoing supervision. It entailed, among other components, a practical 3-day training on IYCF-E delivered to a selected group of health facility staff identified by facility management as IYCF focal persons. The training curriculum followed the recommendations and standards of the OG-IFE. These IYCF focal persons were responsible for leading IYCF counseling, providing onsite mentorship to colleagues, and ensuring adherence to standard counseling protocols and referral pathways.

To extend the reach of the intervention, a 9-h orientation on IYCF-E was conducted for a broader group of facility-based health workers from the same institutions. This shorter course aimed to equip general staff with the essential knowledge and skills to offer basic IYCF support and ensure consistent, accurate information to women and caregivers seeking care.

Between November 2024 and October 2025, FHI 360 supported a total of 123 health facilities across four oblasts (Dnipro, Zaporizhia, Mykolaiv and Odessa), including maternity hospitals, perinatal centers, and primary health care facilities providing antenatal care, postpartum services, well-child consultations, and immunization. In total, 195 health workers designated by facility management participated in the intensive 3-day IYCF-E training, 145 (75%) of whom were officially appointed as primary IYCF focal persons. In addition, 660 health workers from the same health facilities completed the 9-h IYCF-E orientation sessions.

From November–October 2025, health facility IYCF focal persons delivered 4,359 individual counseling sessions across health facilities in four the conflict-affected oblasts: Dnipropetrovska (2,233,51%), Odeska (712,16%), Mykolaivska (795,18%), Zaporizka (619,15%). Many sessions occurred under challenging conditions of displacement and intermittent power and raid alerts.

#### 3.8.2 Client reach and feeding outcomes

A total of 535 women **(13%)** received two or more individual IYCF counseling sessions all women with infants < 6 months of age. Among the 535 mothers with follow-up, exclusive breastfeeding (EBF) rose from 60.1 % to 69.2 %, and formula-only and breastfeeding plus formula feeding declined from 5% to 4.2% and from 17.4% to 14.6% respectively and other milks from 10.6% to 5.5%. The largest EBF gains occurred in Dnipropetrovska and Odeska, where mentorship density was highest.

### 3.9 Integration of mental-health and psychosocial support

MHPSS screening occurred in 35 % (1,654) of counseling sessions. Forty-six mothers required specialized referral; 147 were referred to pediatric, gynaecologic, or rehabilitation services. All were tracked via KoBoToolbox, ensuring continuity. Integrating MHPSS within IYCF counseling demonstrated the **feasibility of holistic maternal-infant care during emergencies**.

### 3.10 Mentorship system and capacity strengthening

A structured mentorship framework paired newly trained counselors with master trainers across Dnipropetrovska, Mykolaivska, Odeska, and Zaporizka. Between March–October 2025, 692 mentorship visits were completed, rising from 7 in March to 158 in September, stabilizing at 32 in October. Dnipropetrovska hosted 44% of sessions, Zaporizka 22%, Odeska 18%, Mykolaivska 16%.

Mentorship focused on observation, feedback, and role-play around:

- Correct application of IYCF-E and BFHI standards
- Adherence to the International Code of Marketing of Breast-milk Substitutes
- Trauma-informed communication and MHPSS integration

By October 2025, mentees were conducting peer-to-peer mentoring, creating a self-sustaining regional network.

### 3.11 Mentorship achievements and system strengthening

Mentorship rapidly enhanced counselor competence and consistency. The number of mentorship sessions increased twenty-fold (March → September), while direct supervision improved both technical accuracy and empathetic communication. Facilities reported more complete records and fewer counseling errors. In oblasts with the densest mentorship (Dnipropetrovska and Odeska), improvements in EBF rates and counseling quality were most pronounced.

### 3.12 Competency progression through mentorship

Eight sequential observation visits (March–July 2025) assessed 44 competencies using a standard checklist. Average adherence increased from 73% to 97%, with >80% of indicators reaching 100% compliance. Table 2 summarizes the **five most illustrative competencies**, arranged from the lowest to highest improvement.

**Table 2:** Progression of counseling competencies across eight mentorship visits (organized from lowest to highest improvement).

Competency	Initial Performance (visit 1–2 avg)	Final Performance (visit 7–8 avg)	Improvement (%)	Remarks
Systematic assessment and analysis of feeding difficulties	90%	100%	+10	Improved ability to identify root causes, prioritize issues, and formulate targeted solutions.
Applying empathetic and non-judgmental communication	88%	100%	+12	Enhanced empathy, active listening, and mother-centered communication reducing directive tendencies.
Use of behavior-change communication and IEC materials	81%	100%	+19	Consistent use of visual and printed tools to reinforce key counseling messages.
Integrating mental health into infant feeding counseling	63%	100%	+37	Marked improvement in embedding psychosocial messages and screening within feeding sessions.
Providing practical breastfeeding support (cup feeding, hand expression, pump use)	57%	100%	+43	Largest gain—counselors became proficient in demonstrating hands-on lactation techniques.

All competencies improved markedly under structured mentorship. The most dramatic progress occurred in practical support and mental-health integration, confirming that regular supervision accelerates both technical and psychosocial skill acquisition.

### 3.13 Competency improvements among counselors

Continuous mentorship converted supervision into experiential learning. Quantitative monitoring across 44 indicators showed sustained improvement, with 25–45 percentage-point gains in targeted competencies.

By July 2025, nearly all counselors demonstrated proficiency in:

- Integrating mental-health messages into IYCF sessions
- Demonstrating cup-feeding and hand-expression techniques
- Using reflective, empathetic communication
- Conducting structured feeding assessments
- Applying IEC and behavior-change tools

One mentor summarized the transformation:

“By the sixth visit, almost every counselor could connect a mother's emotional state to feeding problems and guide her calmly without my help (mentor).”

This evolution demonstrates that mentorship was not simply monitoring, but transformative, confidence-building practice improving both skill and compassion.

### 3.14 Overall impact and scale-up potential

The dual digital-human system produced measurable gains in service continuity, counseling quality, maternal confidence, and EBF outcomes across conflict-affected oblasts.

Implemented within FHI 360-supported facilities with the support from UNICEF Ukraine, the model proved context-appropriate and scalable.

The *Harmony of Parenthood* initiative shows that AI-enabled, human-supervised counseling can sustain maternal and infant nutrition care under crisis conditions. Validated across six oblasts, it is now ready for scale-up in 2026, offering a replicable pathway for integrating digital innovation, mentorship, and empathetic care in humanitarian nutrition systems.

## 4 Discussion

This evaluation shows that a closed-domain, ethically governed AI system can effectively complement human counseling and sustain IYCF-E support during active conflict.

The findings of this evaluation indicate that an AI enabled, human supervised counseling system can feasibly function as a complementary layer within an IYCF-E programme during a period of protracted conflict. Chatbot query distribution and thematic trends reflected caregivers' most common concerns, particularly breastfeeding technique, milk supply, and complementary feeding. These priority topics aligned with the counseling competency framework used in training and mentorship, enabling counselors and mentors to reinforce key areas of support during counseling sessions and strengthen the responsiveness of counseling services. The AI-enabled hybrid model also supported mentoring and counseling by providing caregivers with continuous access to evidence-based information between facility visits. This reinforced counseling messages and allowed mentors and counselors to focus on more complex or individualized support needs during mentorship and counseling sessions. Chatbot interaction trends also provided insights into common caregiver concerns, enabling mentors to better tailor counseling and mentorship activities.

By integrating digital analytics, facility-based counseling data, mentorship competency assessments, and qualitative user feedback, this study provides new evidence on how hybrid digital–human models can strengthen implementation of the Operational Guidance on Infant and Young Child Feeding in Emergencies (OG-IFE), sustain continuity of maternal and infant nutrition services, and enhance health system resilience in conflict-affected contexts. These findings address a critical gap in the evidence base regarding the safe and effective use of artificial intelligence-enabled counseling systems within humanitarian nutrition programmes.

High utilization, strong retention, and consistently high satisfaction suggest that caregivers perceived value in the system's immediacy, clarity, and continuous availability. The themes emerging from user reflections, including appreciation for timely responses, emotional reassurance, and support during periods of stress or restricted mobility, are consistent with global evidence on the role of digital health tools in improving access to information and reducing caregiver anxiety in disrupted settings (40, 41). The large volume of incoming messages, repeated use among many users, and high proportions reporting usefulness and clarity reinforce the acceptability and feasibility of this form of support.

The integration of the chatbot with in-person counseling and mentorship activities appears to have created a coherent hybrid support pathway. Although causal inference cannot be established, the improvements in exclusive breastfeeding among mothers with repeated counseling sessions, the reductions in mixed and formula feeding, and the marked increase in counselor competency scores suggest that the combined digital and human components worked together to strengthen continuity and quality of care. These findings align with global literature indicating that digital health interventions tend to be most effective when used to complement, rather than replace, skilled human support (24, 42, 43). The ability of caregivers to access the chatbot during periods of insecurity or limited facility access likely contributed to its repeated use and high satisfaction, mirroring trends seen in other digital health deployments during crises (44–47).

The intervention also addressed key elements of the Operational Guidance on Infant and Young Child Feeding in Emergencies (9). The chatbot provided accurate, consistent, and Code-compliant information in Ukrainian, fulfilling OG-IFE recommendations related to accessible and reliable communication. The presence of psychosocial support elements within the system aligned with OG-IFE guidance, emphasizing caregiver emotional well-being. The referral mechanism enabling users to transition from digital to facility-based support illustrates the potential of hybrid systems to strengthen coordination between community and health services. The improvements documented through facility mentorship highlight the role of supportive supervision in enhancing counseling quality, a central principle of OG-IFE implementation.

Several factors likely contributed to why the hybrid model functioned effectively. The structured and validated knowledge base provided a reliable foundation for AI generated responses and reduced the risk of contradictory information. The 24-h availability offered caregivers a dependable source of guidance during periods of curfew, displacement, or insecurity. The anonymity of digital interaction may have enabled users to seek support comfortably, particularly when in-person care was not accessible. The escalation process ensured that complex issues received human attention, maintaining clinical appropriateness. The observed gains in mentorship indicators suggest that counselors were increasingly equipped to provide accurate and supportive breastfeeding counseling, reinforcing the impact of follow-up care. Collectively, these system components likely contributed to the positive trends observed in feeding practices and user experience.

Digital ethics and safety considerations were integral to the design. The use of a closed knowledge base, restricted domains, and fallback instructions reflects global recommendations for safe and responsible AI in health (32). Weekly monitoring enabled rapid identification and correction of inaccuracies, and the minimal occurrence of errors suggests that the layered safeguards were effective. The decision not to collect personal identifiers or geolocation data supported user privacy and aligned with WHO principles for responsible AI (32). These measures demonstrate that AI enabled tools can be implemented safely in humanitarian contexts when guided by robust governance frameworks.

This study has several limitations related to the anonymous and voluntary nature of chatbot participation and the interpretation of engagement metrics. During the evaluation period, the chatbot recorded 2,066 unique users, representing individual Telegram accounts that accessed and interacted with the system. Because no personally identifiable information was collected, it was not possible to independently verify whether each account corresponded to a single caregiver or to directly link chatbot use with individual behavioral or health outcomes. While anonymity was essential to ensure user safety and confidentiality in a conflict setting, it introduces potential uncertainty in the precise number and characteristics of individual caregivers represented. In addition, because the chatbot was made openly available to all pregnant women and caregivers in the supported oblasts, no predefined target number of users was established. The observed number of users therefore reflects actual programme uptake rather than a predetermined sample size.

Interpretation of engagement indicators such as bounce rate also requires contextual consideration. The observed bounce rate of 32% reflects users who opened the chatbot but did not initiate a query. In conflict-affected environments, this may reflect external factors such as intermittent internet connectivity, electricity disruptions, air raid alerts, displacement, or exploratory access rather than dissatisfaction with the system. Several measures were implemented to address and mitigate these limitations. Although user anonymity prevented direct verification of individual identities, the system captured unique account-level interaction data, timestamps, and engagement patterns, allowing robust analysis of utilization trends while maintaining user privacy and safety. In addition, the chatbot was integrated within a comprehensive IYCF-E programme that included facility-based counseling, structured mentorship, and supervision, enabling triangulation of digital engagement data with mentorship competency assessments and programme monitoring indicators. Continuous system monitoring and optimisation were conducted throughout implementation to ensure response accuracy, alignment with WHO and national guidance, and usability. These measures strengthened the reliability of the findings and allowed appropriate interpretation of feasibility, acceptability, and operational contribution, while avoiding over interpretation of population-level impact or causal effectiveness.

Although only one hallucination event was detected, it is possible that minor inaccuracies went unreported. These considerations should be taken into account when interpreting the results.

Future research should explore comparative effectiveness between digital-only, hybrid, and in-person support models through experimental or quasi-experimental designs. Investigations into predictors of digital engagement, such as caregiver characteristics, stress levels, and displacement status, would support refinement of content and outreach strategies. Cost-effectiveness analysis is needed to understand the value of hybrid models relative to traditional approaches in emergency or resource-limited settings. Research across other humanitarian contexts would clarify scalability and cultural adaptability. Finally, further examination of AI governance, risk mitigation, and content adaptation processes would inform ethical expansion of similar systems in diverse emergencies.

Long-term success depends on integration into government strategies, interoperability with national data systems, and consistent funding (46, 47). The Harmony of Parenthood initiative generated actionable data for emergency responders and local authorities, revealing peaks in anxiety-related queries during bombardments and informing adaptive communication and service planning. Such data-driven feedback loops exemplify WHO's vision for digital health systems that enable learning and responsive care in fragile settings (46).

The Ukrainian context also underscores the need to adapt IYCF-E programming to high- and middle-income emergencies. While global guidance has largely been developed with low-income settings in mind, humanitarian crises in middle and higher-income contexts pose distinct challenges, including market saturation of CMF, entrenched medicalised birth practices, and lower baseline breastfeeding prevalence (21). In such environments, emergency responses must combine regulatory enforcement, psychosocial support, and digital outreach to counter misinformation and sustain breastfeeding. This aligns with the IFE Core Group, WHO and UNICEF's call for tailored, context-specific IYCF-E strategies and regular assessment of needs, behaviors, and access to IYCF support in line with the International Code of Marketing of Breastmilk Substitutes and the OG-IFE (8, 9, 48–51).

Globally, IYCF-E remains underfunded (52) and insufficiently prioritized despite its proven lifesaving impact (53). The Harmony of Parenthood experience, combining AI-assisted counseling and structured mentorship, provides a scalable, ethically governed model that can strengthen emergency preparedness and bridge this implementation gap.

In conclusion, this initiative demonstrates that digital innovation, when grounded in humanitarian ethics and reinforced by skilled human support, can strengthen the continuity and quality of IYCF services in emergencies. By operationalising the OG-IFE, the Code, and BFHI within an AI-assisted mentorship model, FHI 360 and UNICEF contributed to maintaining breastfeeding support amid war. This experience offers a replicable framework for humanitarian actors and governments to localize IYCF-E capacity, ensure compliance with international standards, and extend equitable access to care for mothers and infants during crises. Protecting, promoting, and supporting IYCF-E remains a lifesaving priority, one that can be advanced through innovative, evidence-based, and ethically designed digital–human partnerships.

## 5 Conclusion

This study demonstrates that an AI-enabled, human-supervised counseling system is feasible, safe, and acceptable for supporting Infant and Young Child Feeding in Emergencies (IYCF-E) during active conflict. High user engagement, sustained utilization, and positive user feedback indicate that caregivers valued the platform as a reliable source of timely, evidence-based guidance, particularly when access to facility-based services was disrupted. The integration of the chatbot within a broader programme of training, mentorship, and referral strengthened continuity of counseling and contributed to improvements in counseling competency and breastfeeding practices among supported caregivers.

These findings show that ethically governed, closed-domain AI tools can effectively complement skilled human support and enhance continuity and quality of IYCF-E services in humanitarian settings. Hybrid digital–human models offer a practical and scalable approach to strengthening access to accurate counseling and maintaining essential maternal and infant nutrition services during emergencies.

These features suggest strong potential for replication and adaptation in other crises, provided that digital tools remain embedded within coordinated, human-centered IYCF-E systems. At the same time, the findings underscore the broader need for transformative investment in maternal and child nutrition during emergencies. Progress in IYCF-E will remain limited unless donors, governments, and humanitarian organizations shift from short-term, fragmented initiatives to long-term, system-strengthening approaches. As conflicts, displacement, and climate-related shocks intensify globally, protecting maternal and young child nutrition must move from the periphery to the center of humanitarian action. The global community can no longer afford to underinvest in the foundational practices that sustain life. Breastfeeding and complementary feeding protect nations, strengthen resilience, and reduce preventable deaths. The model presented in this study offers a scalable framework that integrates digital technology, mentorship, and community-based counseling, providing governments, UN agencies, and partners with a practical approach for strengthening IYCF-E preparedness and ensuring equitable access to life-saving nutrition services in future emergencies.

## Data Availability

The datasets generated and/or analyzed during this study are available from the corresponding author upon reasonable request, in accordance with FHI 360 ethical and data protection requirements.
